# Joseph Lister and the performance of antiseptic surgery

**DOI:** 10.1098/rsnr.2013.0028

**Published:** 2013-05-22

**Authors:** Michael Worboys

**Affiliations:** Centre for the History of Science, Technology and Medicine and Wellcome Unit for the History of Medicine, University of Manchester, Manchester M13 9PL, UK

**Keywords:** Lister, surgery, antisepsis, performance

## Abstract

This article highlights a neglected feature of Joseph Lister's work, namely how, in addition to promoting germ theories and the principles of the antiseptic system, he also devoted much time and effort to communicating the performative aspects of antisepsis and of the many other surgical innovations that he developed. Attention to ‘detail’ and striving for ‘improvement’ were crucial to Listerian practice, and he sought to convey his credo in three main ways: first, his publications aimed at ‘bringing the subject out in the same sort of way as it had been worked out by himself’; second, he set out strict protocols and information on materials and methods, yet also encouraged surgeons to improvise; and third, he made himself an exemplar of a new form of professionalism, which made constancy and vigilance in practice a moral duty for surgeons.

On 1 September 1960, in a medical history series entitled ‘They Made History’, the BBC broadcast a re-enactment (it would now be called a docudrama) of an operation that Joseph Lister performed at King's College Hospital on 26 October 1877.^1^ The patient was Francis Smith, who had fractured his patella (kneecap) a fortnight earlier and had reluctantly agreed to allow Lister to wire together the separated fragments of bone. The programme was based on case notes held at King's College and an address that Lister gave to the Medical Society of London in 1883.^2^ The main message offered to viewers was not the novelty of wiring together broken bones, but the demonstration of how the antiseptic system ‘had removed for ever the threat of hospital disease’.^3^ In fact, the address and article primarily described a novel surgical procedure, promoting antisepsis was secondary, and the published version was rightly included in Lister's collected papers in the section ‘Surgery’, not ‘The Antiseptic System’.^4^ The address, and perhaps inadvertently the television programme, highlighted a feature of Lister's work that has previously been neglected, namely how his publications tried to provide fellow surgeons with the detailed knowledge of materials and technique to perform his methods.^5^ Historians have emphasized how Lister promoted the antiseptic system by stressing ‘principles’ and ‘professionalism’, to which I wish now to add ‘performance’. In this, Lister's work can be seen as ‘performance’ at almost every level identified in recent historical scholarship: demonstrating how to ‘do’ as well as ‘know’, recruiting audiences into ways of seeing and acting, asserting power through the instantiation of ritual and successful practices, and acting out a professional identity.^6^

In recent decades, many studies have shown that the spread of antiseptic methods was neither easy nor rapid, and that exactly how Lister's principles and practices were used varied greatly across medicine.^7^ Moreover, historians are no longer content to assume that antiseptic methods spread because they were ‘successful’ or based on ‘true’ principles. Both these claims are, of course, ahistorical and we now understand that every aspect of the antiseptic system was contested by Lister's contemporaries, not because his critics were ignorant, prejudiced or wrong, but for very good reasons given the surgical knowledge and methods at the time. In relation to the spread and uptake of antiseptic theory and practice, it is no longer sufficient to say that Lister's lectures and publications put his ideas ‘in the air’, whence they spread. Such diffusionist models of the adoption of innovations in medicine have been shown to be inadequate at every level.^8^ In the case of Lister and antisepsis, many surgeons were never persuaded, and others had a ‘pick and mix’ approach. In charting the spread of innovations, historians of medicine now wish to identify the mechanisms and agencies that circulated knowledge and practice. Moreover, they seek to chart how ideas and practices were changed in transit and how they were adopted and adapted in different contexts, along with their meanings, both culturally and technically, to different groups.^9^

Christopher Lawrence and Richard Dixey have demonstrated the importance to Lister of stressing that his new methods were based on principles he took from the germ theory of putrefaction, and they show how he changed both his principles and practices from the 1860s to the 1880s.^10^ Anne Crowther and Marguerite Dupree's recent exploration of the diaspora of Glasgow and Edinburgh medical schools has shown the importance of the embodied knowledge and skills of his students; not just their technical knowledge, but also the way in which Lister was a role model of a gentleman professional.^11^ In this article I add a rereading of Lister's articles and published lectures to show the importance of performance. I challenge the view, promoted by his contemporary critics, and seemingly accepted by many historians, that his writings were unhelpful to his cause and worked against the spread of the antiseptic treatment because they were tedious and confusing. His critics pointed out that he made many modifications to his methods over time, showing that earlier versions must have been defective.^12^ My rereading leads me to side with his contemporary followers and supporters, who maintained that his addresses and articles were detailed and precise to enable his practices to be readily emulated, and that the many changes over time were improvements, showing progress in his methods.^13^ Lister's writings were long-winded in comparison with modern style and taste, but such prolixity was typical of his era and it must be remembered that many of his articles were the transcripts of lengthy clinical demonstrations. Other surgeons wrote similar articles with detailed case histories and technical minutiae; however, Lister was unusual, especially in the 1860s and 1870s, for publishing lengthy pieces and series.

For my argument, a key feature of his published works is that he tried to communicate how to *perform* his methods—especially hand skills, the manipulation of materials and instruments, and teaching the eye to see whether wound healing was good or bad—and to respond creatively to patients' prognoses. Attention to ‘detail’ and striving for ‘improvement’ were crucial to communicating how to perform antiseptic methods in three ways. First, Lister's publications tried to provide readers with all they needed know, plus what they needed to have to hand, to replicate his own practice. His biographer Rickman Godlee, who was his nephew and assistant in London, wrote that Lister's expository style deliberately aimed at ‘bringing the subject out in the same sort of way as it had been worked out by himself’.^14^ Unlike modern workshop manuals, in which the narrator is absent, readers were invited to be at Lister's shoulder and to ‘see’ his performance as he did. This was typical of ‘instruction manuals’ of the time across a range of areas, from chemistry to brewing, in which the personal authority of the writer and their assumed agency was crucial. In cases written up from his surgical demonstrations, this was exactly how it must have been, except that operating theatres in medical schools were large and the audience was at some distance. In ward cases his assistants would have been at his side or actually looking over his shoulder. In all instances, Lister provided step-by-step instructions of what to see and do, when and how to do it, and how to assess outcomes.

Second, and at a more general level, his articles consistently advocated the importance of attention to detail in all aspects of surgical practice, not just in antiseptic methods: he also encouraged problem-solving through improvisation and experiment. Performance had to follow clear protocols, yet surgeons were also told to be flexible and resourceful; Lister often commented favourably on adaptations to his methods made by other surgeons. Third, Lister's professionalism was seen not only in his demeanour and character, but also in his operative performance at every level: no detail was too insignificant for his attention, and yet these myriad details were the basis of his surgical system. Thus, surgeons who did not give constant and vigilant attention to particulars in their practice, or who did not seek to improve performance, were failing in their duty to patients and letting down the profession.

## Antiseptic performance

Joseph Lister's first published account of his use of carbolic acid was in a series of articles in *The Lancet* in the spring of 1867.^15^ The problem he addressed was how to prevent wound infection in compound fractures and abscesses. Surgeons were offered new practices that made the management of compound fractures—where the skin was ruptured, infection likely and healing imperfect—the equivalent of simple fractures, where the broken bones remained entirely an internal injury. The first article featured the now famous case of James Greenlees and those of three other patients. This was the first of four articles, published in successive weeks, in which a further seven cases were described and discussed.^16^ A fifth article on the use of carbolic acid in the treatment of abscesses was added to the series in July 1867.^17^

The cases discussed in the first article were from the previous two years, starting with Greenlees's admission to Glasgow Royal Infirmary on 12 August 1865. Lister wrote that he had delayed publication to refine and validate his methods with more cases. The opening column and a half of the first article set out the principle of protecting wounds from the germs in the atmosphere that caused putrefaction in vulnerable wounds. He then introduced the means of doing this: using carbolic acid as a germicide. The remaining seven columns were given over to the case histories, with narratives of Lister's struggles, typically over months (and with failures as well as successes), to prevent or control putrefaction in wounds. Case histories of wound management dominated the successive articles, although the final article dealt with abscesses, setting out step by step the technical requirements and performance of effective antiseptic treatment. The style of these articles was familiar to readers of *The Lancet*, with case history information on the patient's injury, initial prognosis, treatment and outcome. For example, the fourth article, published on 27 April, was followed in the same journal by an article in the same vein: Thomas Buzzard's account of injuries from railway accidents, in which he gave case histories that illustrated general points he wished to make about ‘railway spine’.^18^

Although the narrative of the patient's progress dominates Lister's articles, there was a subsidiary story in each case of when, how and why Lister had tried different dressings, and over the series—as befits a report of experimental work—the evolution of improvements. For example, in his second case with ‘Patrick F’, the treatment failed, gangrene set in and the leg was amputated. However, Lister emphasized that he had learnt much and suggested further innovations:

While I could not but feel that this case, by its unfortunate issue, might lose much of its value in the minds of others, yet to myself it was perfectly conclusive of the efficacy of carbolic acid for the object in view. At the same time it suggested some improvement in matters of detail. It showed that the acid may give rise to a serous exudation apt to irritate by its accumulation, and therefore that a warm and moist application would be advantageous to soothe the part, and also ensure the free exit of such exuded fluid. At the same time it appeared desirable to protect the crust with something that would retain the volatile organic acid more effectually than oiled silk or gutta-percha, through which it makes its way with the utmost facility. For this purpose a metallic covering naturally suggested itself, and as ordinary tin-foil is unsuitable from its porosity, I employed thin sheet-lead, and afterwards block-tin, such as is used for covering the jars of anatomical preparations, superior to lead on account of the facility with which it can be moulded to any shape that is desired.^19^

In the final article on abscesses, Lister gave similarly detailed instructions of how to prepare and apply dressings, elevating a previously subsidiary element of an operation into a pivotal procedure:

A solution of one part of crystallized carbolic acid in four parts of boiled linseed oil having been prepared, a piece of rag from four to six inches square is dipped in the oily mixture, and laid upon the skin where the incision is to be made. The lower edge of the rag being then raised, while the upper edge is kept from slipping by an assistant, a common scalpel or bistoury dipped in the oil is plunged into the cavity of the abscess, and an opening about threequarters of an inch in length is made, and the instant the knife is withdrawn the rag is dropped upon the skin as an antiseptic curtain, beneath which the pus flows out into a vessel placed to receive it.^20^

Readers were instructed further on the dilutions of carbolic acid, the usage of materials, scalpel techniques and drainage. The remainder of the article is similarly exact and hands-on:

About six teaspoonfuls of the above-mentioned solution of carbolic acid in linseed oil are mixed up with common whitening (carbonate of lime) to the consistence of a firm paste, which is in fact glazier's putty with the addition of a little carbolic acid. This is spread upon a piece of sheet block-tin about six inches square; or common tinfoil will answer equally well if strengthened with adhesive plaster to prevent it from tearing, and in some situations it is preferable, from its adapting itself more readily to the shape of the part affected.^21^

Lister ended with the case of a man who had an abscess that was repeatedly filled by putty infused with carbolic acid, which had to be changed regularly for four months. Yet healing was finally achieved, giving Lister ‘inexpressible happiness’.^22^

Lister published again in *The Lancet* at the end of November 1867: a single article entitled ‘Illustrations of the Antiseptic Treatment, No. 1’, the first in another series.^23^ A footnote stated that, unlike in his first set, he would be ‘untrammelled as to the order in which the subjects are introduced, and shall be at liberty to notice from time to time any improvements that may suggest themselves in the methods of dealing with the various classes of cases—J.L.’. The promised next article on simple incised wounds never appeared, and the reason was never explained. Perhaps it was the hostile reaction to his address at the Annual Meeting of the British Medical Association in Dublin that August, although it is more likely that the series was delayed until the following summer and presented instead in an address to the Medico-Chirurgical Society in Glasgow in May 1868. This address was published in five parts from July 1868 to April 1869.^24^ The final article revealed what became an enduring interest in catgut ligatures. He told his readers that he always carried round with him ‘a small silver bottle with well-fitting screwed top …; and this contains two little rods of wood with gut of two sizes wound upon them, together with a few drops of the antiseptic oil.’^25^ The leading Leeds surgeon Thomas Nunneley responded to the series stating that his colleagues had tried Lister's methods and had abandoned them because they did not work.^26^ Lister responded by quoting a contradictory letter he had received from Nunneley's colleague T. Pridgin Teale, stating that surgeons in Leeds had as much confidence as ever in the antiseptic treatment. Teale also showed the linkage principle and performance, observing ‘Any want of success in our *practice* may fairly be attributed to imperfections in carrying out your *rules*’ (emphasis added).^27^

Lister published on antisepsis at this time in two modes: first, clinical studies in which his principles and practices were set out in the context of case histories of individual patients; and second, programmatic statements linked to germ theories, as when he advanced the benefits of antisepsis for the general salubrity of hospitals and the progress of medicine. The two modes were not exclusive: in the programmatic pieces Lister cited groups of cases and statistics, but typically he did not describe performance. Many histories of Listerian antisepsis have tended to focus on the programmatic papers, which tended to prompt the strongest reactions from his critics, and ignore the seemingly mundane and technical clinical articles.

As we have seen, the antiseptic treatment was announced to the world in 1867 through a series of case histories, and Lister described a further 36 in detail over the next decade.^28^ His reports typically began with an account of how the patient arrived on his ward; they then described the treatment and aftercare, focusing on all aspects of the case and not just on wound management. When the British Medical Association met in Edinburgh in 1875, Lister gave demonstrations in which surgeons were made eye-witnesses to the performance of antiseptically staged operations from start to finish. Indeed, the print version contained updates on the progress that the patients had made since the demonstration.^29^ Some of his publications gave statistics on outcomes before and after his adoption of antiseptic treatment in 1865; however, he assumed, no doubt rightly for the time and his audience, that instructive cases were more persuasive than ‘statistics’ from a relatively small number of patients with variable conditions.

In 1870, when setting out how antiseptic methods could be used in the Franco-Prussian War, Lister produced a ‘plan’ that sought to ‘combine efficiency with the simplicity and facility of execution essential under such circumstances’.^30^ He recommended washing wounds with a 1 in 20 solution of carbolic acid, removing clots, tying bleeding vessels and finally, at greatest length and detail, dressings. Lister then explained the rationale, further elaborating the materials and techniques necessary to convert his principles into practice.

At times it seems that Lister was prepared to compromise on his principles and allow his methods to be judged on their results. In his address in Edinburgh in August 1875, Lister included the following as a footnote to the published lecture:

If anyone chooses to assume that the septic material is not of the nature of living organisms, but a so-called chemical ferment destitute of vitality, yet endowed with a power of self-multiplication equal to that of the organism associated with it, such a notion, unwarranted though I believe it to be by any scientific evidence, will in a practical point of view be equivalent to a germ theory, since it will inculcate precisely the same methods of antiseptic management. It seems important that this should be clearly understood, because it appears to be often imagined that authors who are not satisfied by the strict truth of germ theory, but substitute for it some other hypothesis, invalidate antiseptic practice, which I must repeat, is not affected in one tittle by this theoretical discrepancy.^31^

Lister was seemingly prepared to compromise on whether germs were the cause of putrefaction, but he insisted that, whatever the cause of wound infection, carbolic acid applied with his methods prevented its development, or halted its progress. His famous statement—‘on this principle I have based a practice’—should not be read as ranking principle above practice, but rather that the two were co-constituted in the antiseptic system.

**Figure 1.**
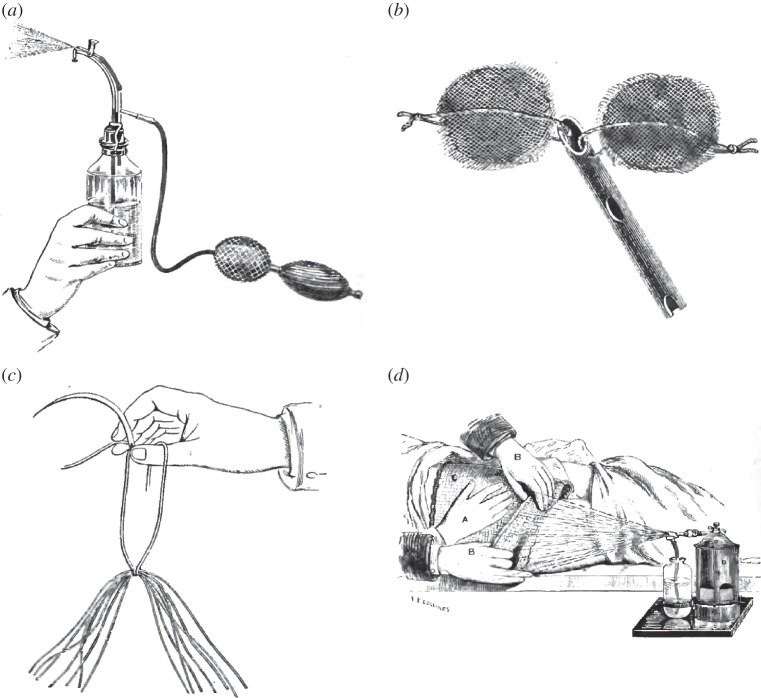
Illustrations from William Watson Cheyne, *Antiseptic Surgery: Its Principles, Practice and History* (Smith, Elder & Co., London, 1882). (*a*) Hand spray producer (p. 54); (*b*) drainage tube with masses of gauze in the loops of thread (p. 77); (*c*) catgut drain ready for insertion (p. 80); (*d*) method of changing a psoas abscess dressing (p. 92).

The first ‘reference-book’ on antiseptic surgery was not published until in 1880, when William MacCormac produced a volume based on the proceedings of the debate held at St Thomas' Hospital, London, in 1879.^32^ The papers and discussion from the meeting took up the first two-thirds of the volume, but in the final third MacCormac dealt with antiseptic practice. According to the review in *British Medical Journal*, the chapters include ‘*complete practical directions*, with clinical illustrations from home and foreign, civil and military surgery [and] *minute directions* as to the management of operations, haemorrhage, (clamp forceps), after dressings, etc.’ [emphasis added]. Attention to detail and performance were a defining feature of the first in-house account of Listerian methods, William Watson Cheyne's *Antiseptic Surgery* published in 1882.^33^ Cheyne had been a student in Lister's classes and became his dresser and his most reliable lieutenant, following ‘the Chief’ to London in 1877. At the core of *Antiseptic Surgery* were chapters on antiseptic materials and methods. His chapters on surgical wound management gave step-by-step instructions on performance, with illustrations of wounds, dressings, instruments and hand skills, all endorsing the importance of developing clinical acumen and technical ability (figure 1). The surgeon's hands were present in some illustrations, again placing the reader at the surgeon's side. Descriptions of performance were vital to Lister's project, as he remarked in 1891: ‘minute details…all illustrate principle’.^34^

## Surgical performance

Joseph Coats, who was a student of Lister's and was appointed to the Chair of Pathology at the Western Infirmary in Glasgow, wrote in 1869 that ‘the chief lesson Lister taught was “carefulness in dressing and operating”’.^35^ More than half a century later, Henry Dobie, who was Lister's dresser and clerk in 1877–78, recalled that his Chief was ‘a master of detail in his work’. He went on to tell how much other than antisepsis he had learnt: ‘I have often found myself saying in my own hospital practice, Lister taught me this or that; for example, to tear a calico bandage, to work with gauze and collodion in single layers, to syringe an ear.’^36^ Throughout his career Lister continued his interest in the technical minutiae of wound management. He published on catgut ligatures in 1869 and 1881, and on how to prepare corrosive sublimate in 1884.^37^ He published again on catgut ligatures in 1908 and 1909, long after he had been lionized by the medical profession and awarded his baronetcy.^38^ Such publications, in the highest-profile journals, signalled yet again to surgeons that in the performance of good surgery no detail should be ignored.

Crowther and Dupree have also pointed to the importance of performance in Lister's classes, noting that ‘By the mid-1860s his operations were the main way of teaching his antiseptic technique.’^39^ Laboratory experiments were also included, with demonstrations of atmospheric germs causing putrefaction, of spontaneous generation failing to occur, and of the pathology of wounds.^40^ Some of his best known lectures interwove laboratory experiments with clinical case histories, as in his address to the Medico-Chirurgical Society of Glasgow in 1868.^41^ At this time, for Lister and opponents of spontaneous generation such as John Tyndall, every successful antiseptic dressing and operation was a refutation of the doctrine of life developing *de novo*.^42^ Besides confirmation of the principles underlying antisepsis, the experiments would have had other meanings for Lister's audience and readers. They indicated that the observational acuity and accuracy required in the laboratory should also be a feature of the clinic and especially the operating theatre. Furthermore, they indicated that ‘experiment’, in the sense of trying new methods, was legitimate. Indeed, experiment based on ‘true’ principles was bound to lead to improvement in surgical outcomes. Thomas Schlich has recently shown that such claims were largely rhetorical, because Lister's antiseptic practices were not based on specific laboratory findings or tests.^43^

**Figure 2.**
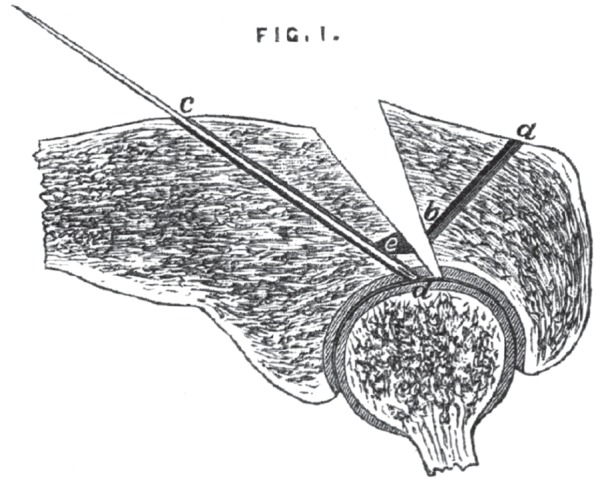
Diagram of an oblique fracture (referred to as Fig. 1 in the quotation in the text). (From Joseph Lister, ‘An Address on the Treatment of Fracture of the Patella, Delivered at the First Meeting of the Session of the Medical Society of London’, *Br. Med. J.* **ii**, 855–860 (1883), at p. 855.)

In the operation to wire the fractured patella reported in 1883, Lister was typically performative in his presentation. Around an illustration of a fractured shoulder socket (figure 2), he presented his techniques in detail:^44^

On the 28th of the month, I made a longitudinal incision, exposing the site of the fracture, and, at the same time, bringing into view the articular surface of the humerus; and, having pared away the fibrous material from the fractured surfaces, I proceeded to drill the fragments, with a view to the application of the suture. The fracture was oblique from before backwards, as indicated by this diagram. I found no difficulty, with the proximal fragment, in making the drill appear upon the fractured surface at a little distance from the cartilage, (see Fig. 1) but with the other fragment the obliquity of the position in which the drill had to be placed was so great that, instead of the drill emerging at the fractured surface, as I had intended, I found it had entered into the substance of the humerus (d, Fig. 1).

I therefore withdrew the drill, and substituted for it a needle (c d), passing the eyed end in first. Then, with a gouge, I excavated an opening (e) upon the fractured surface, opposite to the drill-hole (b) on the other surface, until the needle was exposed. Withdrawing the needle, I introduced a silver wire in its place, and I had no difficulty, by means of forceps passed into the excavation made by the gouge, in drawing out the wire. I was then able to pass it on through the other drilled opening, and thus the two fragments were brought into apposition. The ends of wire were twisted together and left projecting in the wound.^45^

This is another fine example of the presence of the narrator, with the author's authority confirmed by the successful outcome of the case—‘I afterwards had the satisfaction of learning that he was wielding the hammer in an iron shipbuilding yard with his former energy.’

## Professional performance

Crowther and Dupree have shown that to his students Lister was a ‘moral exemplar’ and that socially he was the epitome of a gentleman. They quote James Pringle's obituary, which observes that Pringle, like Lister, fulfilled his voluntary hospital duties with ‘unfailing regularity’ and ‘that even after a long and tiring day in the out-patient department his work would be completed with the thoroughness which was characteristic of the man.’^46^ Lister's attention to detail in his work was captured in descriptions of his character, for which perhaps the most frequently used term was ‘earnest’. In fact, ‘earnest’ and ‘earnestness’ were two of ‘the Chief's’ own favourite terms, which he used repeatedly in his publications to indicate the frame of mind required to perform effective antiseptic surgery. For example, in 1870 he stated that antiseptic techniques ‘require earnest practical study until their employment has become habitual and instinctive.’^47^

Principle, professionalism and performance were not separate features of Lister's antiseptic system, they were co-constituted and synergistic in its practice and its promotion. Historians have tended to focus on how the antiseptic *treatment* of the 1860s became the antiseptic *system* of the 1870s, and then how principles and practices were adapted to the new principles of the new bacteriology and the practices of asepsis. Yet, for most of this period, for his fellow surgeons he was offering new ways of performing operations, new materials, instruments and techniques, which required ways of doing, technical and social, as well as ways of knowing.^48^

The problems of turning words into deeds are known to anyone who has tried to follow the most basic food recipe and have been an issue in medical educations for centuries.^49^ Clinical teaching has always had to be ‘hands-on’ in developing skills and tuning the five senses, with the ‘art’ of medicine and tacit understandings as important as ‘science’ and formal knowledge.^50^ In the particular case of surgeons, ‘hands-on’ has quite literally meant that—how to cut and sew—and beyond this how to handle tissues and organs, use instruments and machines, and deal with patients and staff. Crowther and Dupree have shown how Lister worked with local instrument makers and suppliers to fashion new equipment. My reading of Lister's published lectures and articles is that, as an experienced and effective clinical teacher himself, he was only too aware of the problems of communicating surgical methods and that he sought to deal with this by including the performative aspects of antiseptic and other methods in his writings.

## Notes

1 Frederick F. Cartwright, *Joseph Lister: the man who made surgery safe* (Weidenfeld & Nicolson, London, 1963), pp. 82–92.

2 Joseph Lister, ‘An Address on the Treatment of Fracture of the Patella, Delivered at the First Meeting of the Session of the Medical Society of London’, *Br. Med. J.* **ii**, 855–860 (1883).

3 Cartwright, *op. cit.* (note 1), p. 93.

4 Joseph Lister *The collected papers of Joseph, Baron Lister* (Clarendon Press, Oxford, 1909), pp. v–vii. [Hereafter *Collected papers*.]

5 Thomas Schlich, ‘Farmer to industrialist: Lister's antisepsis and the making of modern surgery in Germany’, *Notes Rec. R. Soc.* **67** (this issue) (http://dx.doi.org/rsnr.2013.0032).

6 See the special ‘Focus’ section in the journal *Isis* entitled ‘Placing performance’, *Isis* **101**, 775–828 (2010), and especially the introduction by Iwan Rhys Morus, *Isis* **101**, 775–778 (2010); M. Worboys, ‘Practice and the science of medicine in the nineteenth century’, *Isis* **102**, 109–111 (2011).

7 Lindsay Granshaw, ‘“Upon this principle I have based a practice”: the development of antisepsis in Britain, 1867–90’, in *Medical innovation in historical perspective* (ed. John V. Pickstone), pp. 17–46 (Macmillan, Basingstoke, 1992); Michael Worboys, *Spreading germs: disease theories and medical practice* (Cambridge University Press, 2000), pp. 73–107 and 150–172.

8 Jennifer Stanton, *Innovations in health and medicine: diffusion and resistance in the twentieth century* (Routledge, London, 2002); Pickstone (ed.), *op. cit.* (note 7), *passim*.

9 James. A. Secord, ‘Knowledge in transit’, *Isis* **95**, 654–672 (2004).

10 Christopher Lawrence and Richard Dixey, ‘Practising on principle: Joseph Lister and the germ theories of disease’, in *Medical theory, surgical practice* (ed. Christopher Lawrence), pp. 153–215 (Routledge, London, 1993).

11 M. Anne Crowther and Marguerite W. Dupree, *Medical lives in the age of surgical revolution* (Cambridge University Press, 2007), pp. 96–110, 221–247 and 367–368.

12 William Watson Cheyne, ‘Reminiscences of “the Chief”’, in *Joseph, Baron Lister: centenary volume, 1827–1927* (ed. Arthur Logan Turner), p. 128 (Oliver & Boyd, London, 1927).

13 Lister celebrated changes to his methods in a series of seven articles published in 1875. Joseph Lister, ‘On Recent Improvements in the Details of Antiseptic Surgery’, *Lancet* **i**, 365–367, 401–402, 434–436, 468–470, 603–605, 717–719, 787–789 (1875). Edward R. Howard, ‘Joseph Lister: his contributions to early experimental physiology’, *Notes Rec. R. Soc.* **67** (this issue) (http://dx.doi.org/rsnr.2013.0029).

14 Rickman J. Godlee, *Lord Lister* (Clarendon Press, Oxford, 1924), p. 189.

15 Joseph Lister, ‘On a New Method of Treating Compound Fracture, Abscess, &c., with Observations on the Conditions of Suppuration’, *Lancet* **i**, 336–339 (1867).

16 *Op. cit.* (note 15), pp. 357–359, 387–389 and 507–509.

17 Joseph Lister, ‘On a New Method of Treating Compound Fracture, Abscess, &c.’, *Lancet* **ii**, 95–96 (1867).

18 Ralph Harrington, ‘On the tracks of trauma: railway spine reconsidered’, *Soc. Hist. Med.* **16**, 209–223 (2003).

19 Lister, *op. cit.* (note 15), p. 338.

20 Lister, *op. cit.* (note 17), p. 95.

21 Ibid.

22 Ibid.

23 Joseph Lister, ‘Illustrations of the Antiseptic System’, *Lancet* **ii**, 667–668 (1867).

24 Joseph Lister, ‘An Address on the Antiseptic System of Treatment in Surgery, Delivered before the Medico-Chirurgical Society of Glasgow’, *Br. Med. J.* **ii**, 53–57, 101–102, 461–463, 515–517 (1868); **i**, 301–304 (1869).

25 *Br. Med. J.* **i**, 304 (1869).

26 Thomas Nunneley, ‘Address in Surgery’, *Br. Med. J.* **ii**, 152 (1869).

27 Joseph Lister, ‘Mr. Nunneley and the Antiseptic Treatment’, *Br. Med. J.* **ii**, 256–257 (1869).

28 The cases were counted from the articles in Part III, ‘The Antiseptic System’, in *Collected papers*, pp. 1–371.

29 Joseph Lister, ‘Demonstrations of antiseptic surgery before members of the British Medical Association’, *Edinb. Med. J.* **21**, 193, 481 (1875–76).

30 Joseph Lister, ‘A Method of Antiseptic Treatment Applicable to Wounded Soldiers in the Present War’, *Br. Med. J.* **ii**, 243–244 (1870).

31 Lister, *op. cit.* (note 13), p. 435.

32 William MacCormac, *Antiseptic Surgery* (Smith, Elder & Co., London, 1880).

33 William Watson Cheyne, *Antiseptic Surgery: Its Principles, Practice and History* (Smith, Elder & Co., London, 1882).

34 *Collected papers*, p. 346.

35 Quoted in Crowther and Dupree, *op. cit.* (note 11), p. 110.

36 Henry Dobie, ‘Reminiscences of “the Chief”’, in Turner (ed.), *op. cit.* (note 12), p. 154.

37 Joseph Lister, ‘An Address on Corrosive Sublimate as a Surgical Dressing: Delivered at the Opening Meeting of the Medical Society of London’, *Br. Med. J.* **ii**, 803–808, 1018 (1884); ‘Observations on the Ligature of Arteries under the Antiseptic System’, *Lancet* **i**, 451–455 (1869); ‘An Address on the Catgut Ligature, Delivered before the Clinical Society of London’, *Clin. Soc. Trans.* **14**, 43–63 (1881).

38 Joseph Lister, ‘Note on the Preparation of Catgut for Surgical Purposes’, *Br. Med. J.* **i**, 125–126 (1908); ‘On Sulpho-chromic Catgut’, *Br. Med. J.* **i**, 245 (1909).

39 Crowther and Dupree, *op. cit.* (note 11), p. 103.

40 *Ibid*., pp. 103–106.

41 Lister, *op. cit.* (note 24).

42 James Strick, *Sparks of life: Darwinism and the Victorian debates over spontaneous generation* (Harvard University Press, Cambridge, MA, 2002), pp. 129–156; Worboys, *op. cit.* (note 7), pp. 86–90.

43 Thomas Schlich, ‘Asepsis and bacteriology: a realignment of surgery and laboratory science’, *Med. Hist.* **56**, 308–343 (2012).

44 Lister, *op. cit.* (note 2), p. 855.

45 *Ibid*.

46 Crowther and Dupree, *op. cit.* (note 11), p. 370.

47 Joseph Lister, ‘Further evidence regarding the effects of the antiseptic system of treatment upon the salubrity of a surgical hospital’, *Lancet* **ii**, 287–288 (1870).

48 John V. Pickstone, ‘Sketching together the modern histories of science, technology, and medicine’, *Isis* **102**, 123–133 (2011).

49 For a modern perspective on this problem, see Roger L. Kneebone, ‘Practice, rehearsal, and performance—an approach for simulation-based surgical and procedure training’, *J. Am. Med. Assoc.* **302**, 1336–1338 (2009).

50 William F. Bynum and Roy Porter, *Medicine and the five senses* (Cambridge University Press, 1993); Christopher Lawrence, ‘“Incommunicable knowledge”: science, technology and the clinical “art” in Britain, 1850–1910’, *J. Contemp. Hist.* **20**, 503–520 (1985).

